# Programmable Metal–Organic Framework Biointerfaces Against Pathogens

**DOI:** 10.3390/biology15131053

**Published:** 2026-07-01

**Authors:** Jiewen Hou, Xinzhe Song, Kaiyang Zhang, Xuehao Huo, Xinhao Sun, Kerun Zhang, Ning Wen, Di Liu, Liwei Chen, Chuncheng Xu, Yen Leng Pak, Zhenbin Guo, Huizi Huang, Ruodan Han

**Affiliations:** 1 College of Biological and Chemical Engineering, Qilu Institute of Technology, Jinan 250200, China; 2 Shandong Provincial Key Laboratory of Green and Intelligent Building Materials, University of Jinan, Jinan 250022, China; 3 Department of Pharmaceutical Engineering, School of Life and Health Sciences, Huzhou College, Huzhou 313000, China; 4 Advanced Technology Research Institute, Beijing Institute of Technology, Jinan 250300, China

**Keywords:** metal–organic frameworks (MOFs), biointerfaces, programmable anti-pathogen systems, pathogen surveillance, immune engineering, biomimetic delivery, biosensing, antiviral therapeutics

## Abstract

Emerging viral diseases continue to pose major challenges to global health, creating demand for materials that can support pathogen control, diagnosis, and therapy. Owing to their tunable structures and versatile biointerfaces, metal–organic frameworks (MOFs) have attracted increasing attention in anti-pathogen applications. While previous studies have often focused on individual functions such as catalysis, biosensing, or drug delivery, a broader perspective on the functional development of MOF-based systems remains limited. In this Review, we summarize recent advances in MOF-enabled pathogen inactivation, diagnostic biosensing, host-directed intervention, and virus-inspired therapeutic platforms. Emerging opportunities in antiviral drug discovery and artificial intelligence-assisted materials design are also discussed. In addition, key challenges associated with structural stability, biosafety, scalable fabrication, and clinical translation are highlighted. This Review provides an overview of current progress and outlines perspectives for the future development of MOF-based anti-pathogen technologies.

## Simple Summary

Metal–organic frameworks (MOFs) are porous materials that can be tailored for a variety of biomedical applications. Although initially investigated mainly for antimicrobial and antiviral inactivation, recent studies have shown that MOFs can also support pathogen detection, immune regulation, and therapeutic delivery. This Review summarizes recent progress in MOF-based anti-pathogen systems from these different perspectives and discusses emerging opportunities in drug discovery and artificial intelligence-assisted design. Current challenges associated with stability, biosafety, and clinical translation are also highlighted. By providing an overview of these developments, this Review aims to offer insights into the future development of MOF-enabled strategies against infectious diseases.

## 1. Introduction

Evolution of MOF anti-pathogen technologies from direct pathogen killing to multifunctional biological defense systems. The framework illustrates the progression from pathogen inactivation, surveillance and diagnosis, and host-directed intervention to virus-inspired therapeutic platforms and emerging intelligent design strategies, highlighting the expanding role of MOFs as integrated biointerfaces across the anti-pathogen process.

Emerging and re-emerging viral diseases continue to pose a persistent challenge to global public health [1,2,3,4]. Recent outbreaks of influenza viruses, coronaviruses, hemorrhagic fever viruses, and other emerging pathogens have demonstrated how rapidly infectious agents can spread across geographical and species boundaries, placing enormous pressure on healthcare systems and socioeconomic infrastructures [5,6,7]. Although substantial progress has been achieved in vaccine development, antiviral therapeutics, and molecular diagnostics, effective pathogen control remains difficult because viruses constantly evolve through mutation, immune escape, environmental adaptation, and host switching [8,9,10,11]. Consequently, there is an increasing demand for next-generation anti-pathogen technologies that can operate beyond single-function interventions and address multiple stages of the infection cycle in a coordinated manner [12,13,14].

Conventional anti-pathogen strategies are typically developed as independent technological modules [15,16,17]. Disinfectants and catalytic materials are designed to inactivate pathogens, and biosensors are optimized for detection and surveillance, while therapeutic systems primarily focus on drug delivery or immune regulation [18,19]. Although each approach has achieved considerable success individually, their functional separation often limits the ability to respond to the dynamic interplay among pathogens, host biological systems, and environmental factors. As infectious diseases become increasingly complex and multidimensional, the development of integrated platforms capable of simultaneously supporting pathogen elimination, diagnosis, therapeutic intervention, and biological regulation has emerged as an important frontier in biomedical research [20,21,22].

Metal–organic frameworks (MOFs), a class of crystalline porous materials assembled from metal nodes and organic linkers, have attracted growing attention in this context [23,24,25,26]. Owing to their exceptionally high surface areas, tunable pore structures, modular compositions, and programmable chemical functionalities, MOFs offer a unique materials platform for engineering biological interactions across molecular, cellular, and tissue scales. Beyond their traditional roles in gas storage, separation, and catalysis [27,28,29], MOFs have increasingly been explored for biomedical applications, where their structural versatility enables the integration of catalytic activity, molecular recognition, cargo delivery, and biointerface regulation within a single material system [30,31,32,33].

Compared with other anti-pathogen material platforms, including inorganic nanoparticles, polymeric nanocarriers, liposomes, hydrogels, and covalent organic frameworks (COFs), MOFs possess several distinctive advantages. Their crystalline architectures provide atomically tunable coordination environments, enabling precise regulation of catalytic activity, molecular recognition, and guest–host interactions. In addition, the coexistence of high porosity, structural modularity, and chemical programmability allows multiple functions—including pathogen inactivation, biosensing, therapeutic delivery, and immune modulation—to be integrated within a single material platform. These characteristics distinguish MOFs from many conventional systems that are often optimized for only one or two specific functions. However, MOFs also face important limitations, including framework instability under certain physiological conditions, potential concerns regarding long-term biosafety, and challenges associated with scalable manufacturing and regulatory standardization. Consequently, the growing interest in MOFs does not imply that they will replace existing anti-pathogen technologies; rather, they provide a complementary materials platform whose unique combination of structural programmability and multifunctional integration offers new opportunities for addressing complex infectious disease challenges.

Importantly, recent advances reveal that the role of MOFs in antiviral and anti-pathogen research has undergone a profound conceptual transformation [34,35,36]. Early investigations largely focused on direct pathogen inactivation through reactive oxygen species (ROS) generation, metal-ion release, or photocatalytic disinfection [37,38,39]. However, contemporary MOF systems increasingly exhibit far more sophisticated biological functionalities. Rationally engineered nanozymes can selectively disrupt viral envelopes and proteins through biomimetic catalytic pathways; MOF-enabled biosensors can achieve ultrasensitive pathogen detection through synergistic signal amplification mechanisms; immunomodulatory MOFs can reshape host immune responses and regulate disease microenvironments; and virus-inspired MOF architectures can emulate natural infection and intracellular trafficking processes to enhance therapeutic delivery [40,41,42]. More recently, artificial intelligence (AI)-assisted design strategies have begun to accelerate the discovery and optimization of MOF platforms under complex biological constraints [43,44]. Collectively, these developments indicate that MOFs are evolving from passive antimicrobial materials into multifunctional biointerface systems capable of interacting with pathogens, host cells, immune networks, and biological information flows [45,46,47].

Despite the rapid expansion of this field, current understanding remains highly fragmented. Existing reviews have predominantly examined MOFs within isolated application domains, including antiviral catalysis, biosensing, drug delivery, nanozymes, or general biomedical applications [48,49,50]. While these studies have provided valuable technological insights, they typically categorize MOFs according to material properties or individual application scenarios and therefore offer limited understanding of how the functional roles of MOFs have evolved across the anti-pathogen process. To the best of our knowledge, although previous reviews have discussed some of these topics individually, their integration within a unified systems-oriented framework remains limited. Consequently, the broader transition of MOFs from single-function antimicrobial materials to multifunctional anti-pathogen systems remains insufficiently articulated. Addressing this gap is important because the next generation of anti-pathogen technologies increasingly requires coordinated interactions among pathogen control, diagnosis, therapeutic intervention, and biological regulation rather than isolated functions.

**Figure 1:**
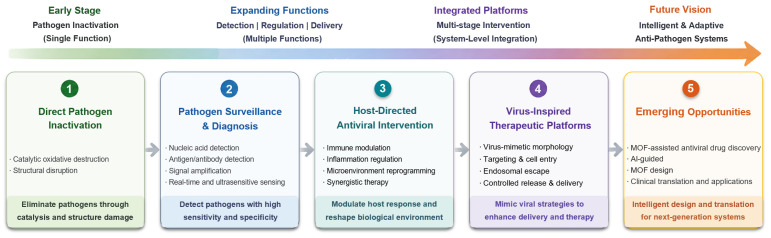
Conceptual evolution of MOF anti-pathogen systems.

In this Review, we address this question by proposing a systems-oriented framework for understanding the evolution of MOF-based anti-pathogen technologies (Figure 1). Rather than classifying studies according to material composition or individual application scenarios, we organize recent advances based on their functional roles throughout the anti-pathogen process. Specifically, we discuss: (i) direct pathogen inactivation through catalytic destruction and structural disruption; (ii) pathogen surveillance and diagnosis through advanced biosensing platforms; (iii) host-directed antiviral intervention via immune modulation and therapeutic reprogramming; (iv) virus-inspired therapeutic systems that emulate biological infection and intracellular delivery pathways; and (v) emerging opportunities involving MOF-assisted antiviral drug discovery, AI-guided materials design, and clinical translation. By integrating these diverse developments into a unified conceptual framework, we aim to elucidate the design principles underlying the transformation of MOFs from pathogen-killing materials into adaptive anti-pathogen systems, thereby providing a conceptual framework for future research and development of MOF-based anti-pathogen technologies.

## 2. Direct Pathogen Inactivation

### 2.1. Catalytic Oxidative Destruction

Catalytic oxidative inactivation represents one of the most extensively explored antiviral mechanisms in MOF-based disinfection systems. Unlike conventional disinfectants that rely on direct chemical toxicity, MOFs can act as catalytic platforms that continuously convert environmental oxygen or endogenous substrates into reactive oxidative species, thereby inducing irreversible damage to viral proteins, lipid envelopes, and nucleic acids. Owing to their tunable coordination environments, adjustable electronic structures, and enzyme-mimicking catalytic activities, MOFs provide unique opportunities to engineer highly efficient antiviral catalysts with controllable reactivity and environmental adaptability.

**Figure 2:**
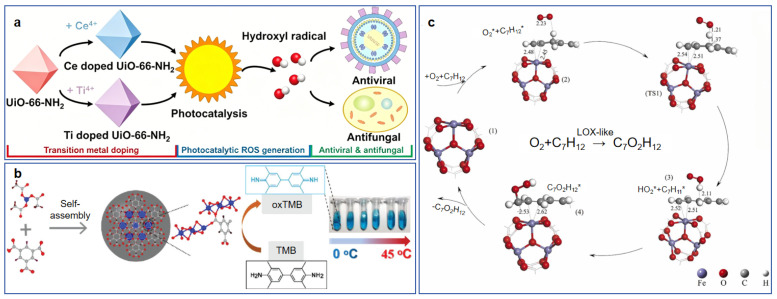
Catalytic antiviral disinfection mechanisms enabled by MOFs. (**a**) Visible-light-responsive Ti/Ce-doped UiO-66-NH_2_ photocatalysts with engineered band structures promote ROS generation through the •OOH → H_2_O_2_ → •OH pathway, leading to efficient viral inactivation under white-light irradiation [51]. (**b**) Synthesis of the manganese-based nanozyme nMnBTC and its oxidase-mimicking catalytic activity across a broad temperature range, enabling low-temperature antiviral disinfection [52]. (**c**) Biomimetic lipoxygenase-like catalytic mechanism of histidine-coordinated Fe-MOF nanozymes, showing selective dioxygenation of unsaturated lipid substrates and consequent disruption of viral envelopes. Bond distances are shown in Å [53].

A major strategy involves the rational regulation of MOF electronic structures to enhance photocatalytic reactive oxygen species (ROS) generation under biologically relevant irradiation conditions. As illustrated in Figure 2a, Yu et al. engineered Ti/Ce-doped UiO-66-NH_2_ through simultaneous metal doping and ligand functionalization, enabling precise bandgap modulation from 1.70 to 2.8 eV and extending photocatalytic activity into the visible-light region. The optimized 20% Ti/Zr-UiO-66-NH_2_ exhibited nearly complete recovery of HSV-infected cells within 120 min, which was consistent with the reported antiviral activity of the photocatalytic system. Mechanistic investigations revealed an unusual photocatalytic cascade involving sequential conversion of O_2_ to •OOH, H_2_O_2_, and ultimately highly reactive •OH radicals (Figure 2a) [51]. Compared with pristine UiO-66-NH_2_ and TiO_2_, the engineered MOF generated substantially higher hydroxyl radical levels, highlighting how molecular-scale band-structure engineering can overcome the ultraviolet dependence that traditionally limits photocatalytic antiviral systems. Importantly, this study demonstrates that catalytic efficiency can be regulated not only through active-site design but also through manipulation of charge-carrier dynamics and ROS conversion pathways.

Beyond light-driven catalysis, MOFs have increasingly been developed as nanozyme platforms capable of sustaining oxidative antiviral activity independent of external irradiation. Inspired by natural oxidases, Chen et al. constructed a manganese-based nanosized MOF (nMnBTC) that functions as a cold-adapted oxidase mimic with remarkable catalytic stability over a broad temperature range (Figure 2b) [52]. Unlike natural enzymes that typically exhibit narrow temperature optima, nMnBTC maintained high catalytic activity from 0 °C to 45 °C and retained antiviral efficacy against H_1_N_1_ influenza virus even at −20 °C [52]. Structural analyses suggested that flexible coordination environments and favorable substrate interactions contribute to its exceptional low-temperature activity. From a translational perspective, such temperature-independent catalytic behavior is particularly attractive for antiviral protection in cold-chain transportation and low-temperature environments, where conventional disinfection technologies often become ineffective.

Despite these advances, several intrinsic limitations of catalytic antiviral systems should be recognized. Most ROS-mediated mechanisms rely on highly reactive oxidative intermediates with limited substrate selectivity, which raises concerns regarding unintended damage to surrounding biomolecules and healthy tissues. In addition, catalytic performance is often influenced by environmental factors, including oxygen availability, pH, light intensity, temperature, and the presence of competing biomolecules, potentially leading to substantial discrepancies between simplified laboratory conditions and complex biological environments. Catalyst deactivation, uncontrolled degradation, and batch-to-batch variability further complicate long-term performance and reproducibility. Moreover, although many catalytic systems demonstrate excellent antiviral activity in vitro, systematic evaluations under physiologically relevant conditions and comprehensive biosafety studies remain relatively scarce. These limitations highlight the need to balance catalytic efficiency with selectivity, stability, and translational feasibility in the design of next-generation antiviral catalysts.

The evolution of MOF nanozymes has further progressed from nonspecific oxidation toward biomimetic catalytic specificity. As shown in Figure 2c, Zhong et al. developed a histidine-coordinated Fe-MOF nanozyme (Fe MOF@His) that mimics the catalytic behavior of natural lipoxygenases (LOXs), enabling selective oxidation of polyunsaturated lipid substrates present in viral envelopes [53]. Unlike traditional peroxidase- or oxidase-like nanozymes that rely on indiscriminate ROS generation, Fe MOF@His exhibited substrate-selective lipoxidase activity and preferentially catalyzed lipid peroxidation reactions resembling those of biological LOX enzymes [53]. This biomimetic mechanism resulted in efficient inactivation of enveloped influenza viruses while maintaining activity across a wide range of temperatures and environmental conditions. Because this strategy relies on selective lipid peroxidation of viral membranes, its antiviral activity is expected to be primarily applicable to enveloped viruses. In contrast, non-enveloped viruses lack lipid envelopes and therefore are unlikely to be susceptible to this mechanism. The transition from generalized ROS production to targeted catalytic disruption of viral membranes provides a more selective antiviral approach while potentially reducing collateral oxidative damage to surrounding biological systems.

Taken together, these studies reveal a clear developmental trajectory in catalytic MOF disinfectants, progressing from photocatalytic ROS amplification toward environmentally adaptive nanozymes and ultimately to biologically inspired catalytic systems with enhanced substrate specificity [54,55,56]. This evolution highlights a broader design principle for next-generation antiviral MOFs: the integration of catalytic efficiency, environmental robustness, and mechanistic selectivity into a single platform capable of achieving effective and sustainable pathogen inactivation [57].

### 2.2. Structural Disruption of Pathogens

Beyond ROS-mediated catalytic inactivation, an emerging strategy in MOF-based disinfection focuses on the direct destruction of essential structural components of pathogens. Rather than relying primarily on oxidative stress, these systems interfere with the physical integrity of viral proteins, lipid envelopes, or surface architectures, thereby rendering pathogens noninfectious. Such approaches are particularly attractive for long-term environmental protection because they can be integrated into protective textiles, coatings, and filtration materials while maintaining activity under challenging operational conditions.

**Figure 3:**
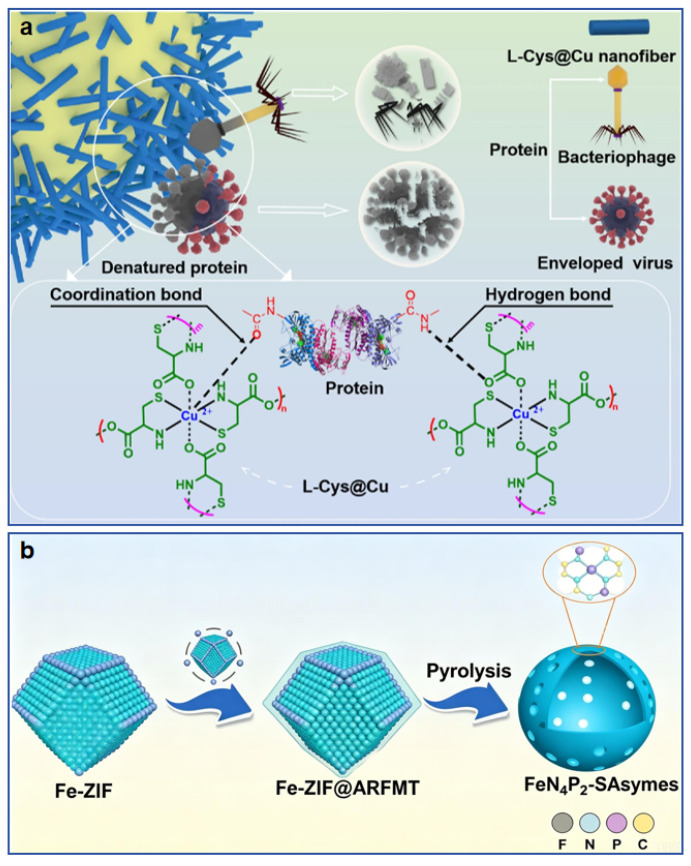
Structural disruption pathways for MOF-based antiviral protection. (**a**) Protein denaturation-mediated viral inactivation by L-Cys@Cu MOF-functionalized fibers [58]. (**b**) Lipid peroxidation-mediated envelope destruction by FeN_4_P_2_ single-atom nanozymes under cold-chain conditions [59].

A representative example is the L-Cys@Cu MOF nanofiber system developed by Xiao et al., in which a copper–cysteine coordination framework was covalently immobilized onto cotton fibers to create durable antiviral fabrics (Figure 3a) [58]. Unlike conventional antiviral coatings that depend mainly on metal-ion release or ROS generation, this work provided direct experimental evidence that MOF nanofibers can inactivate viruses through protein denaturation. Using α-amylase as a model protein, the authors demonstrated that interactions between the L-Cys@Cu framework and protein structures induced conformational disruption, ultimately leading to loss of biological function. This mechanism offers an important conceptual advance because viral infectivity fundamentally depends on the structural integrity of capsid and surface proteins. Once these proteins undergo irreversible denaturation, viral attachment, entry, and replication processes are effectively blocked. Notably, the antiviral activity remained nearly unchanged after extensive mechanical abrasion and repeated washing cycles, highlighting the feasibility of integrating MOF materials into reusable personal protective equipment and healthcare textiles [58].

Whereas protein denaturation targets viral macromolecules, another structural inactivation pathway focuses on the viral lipid envelope. Qin et al. developed FeN_4_P_2_ single-atom nanozymes exhibiting lipid oxidase-like activity across a broad low-temperature range, including 4 °C and −20 °C (Figure 3b) [59]. By introducing phosphorus atoms into the Fe–N–C coordination environment, the electronic structure of the catalytic center was optimized to maintain catalytic activity under cold-chain conditions. The resulting nanozymes selectively catalyzed lipid peroxidation within viral envelopes, leading to membrane destabilization and irreversible loss of infectivity. Importantly, this mechanism proved effective against a wide spectrum of enveloped viruses, including human, swine, and avian coronaviruses as well as multiple influenza A virus subtypes. Since the antiviral activity originates from lipid peroxidation within viral envelopes, this strategy is inherently limited to membrane-containing viruses. Non-enveloped viruses, which lack lipid bilayers, may require alternative inactivation mechanisms targeting capsid proteins or viral genomes [59]. The ability to retain antiviral efficacy at subzero temperatures addresses a major limitation of many conventional disinfectants, whose performance deteriorates dramatically in refrigerated transportation and storage environments.

Although both studies belong to the category of direct structural disruption, they target distinct viral components and therefore illustrate complementary design principles. The L-Cys@Cu MOF system acts primarily through protein destabilization, whereas FeN_4_P_2_ nanozymes destroy the viral lipid envelope through catalytic peroxidation [58,59]. Together, these examples highlight a broader transition in MOF-based disinfection from nonspecific antimicrobial activity toward mechanism-oriented pathogen destruction. Instead of merely generating reactive species, modern MOF platforms are increasingly engineered to attack specific structural vulnerabilities of pathogens, including proteins, membranes, and interfacial assemblies.

## 3. Pathogen Surveillance and Diagnosis

### Biosensing Strategies

**Figure 4:**
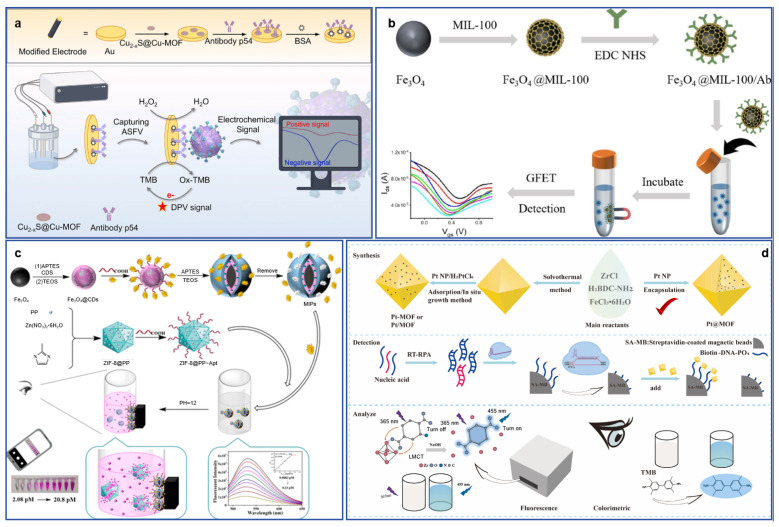
Representative MOF-based diagnostic strategies for viral detection. (**a**) Cu_2-X_S@Cu-MOF heterojunction nanozyme-enabled electrochemical sensing of ASFV [61]. (**b**) Magnetic Fe_3_O_4_@MIL-100-assisted enrichment coupled with GFET detection of SARS-CoV-2 [62]. (**c**) Aptamer-integrated molecularly imprinted ZIF-8 sensor for dual-mode detection of EV71 [63]. (**d**) Pt@MOF-assisted CRISPR biosensing platform for fluorescence and colorimetric detection of norovirus [64].

The rapid and accurate identification of viral pathogens is a prerequisite for effective outbreak control and epidemiological surveillance. However, the intrinsic heterogeneity of clinical samples, low viral titers during early infection stages, and the need for point-of-care operability collectively impose stringent requirements on biosensing platforms, including ultra-high sensitivity, robust selectivity, and signal amplification capability [60]. In this context, MOF-based materials have emerged as versatile interfaces for biosensing, benefiting from their tunable porosity, modifiable surface chemistry, and ability to integrate catalytic, magnetic, and electronic functionalities within a unified platform. As shown in Figure 4a, Zhao et al. developed a heterojunction nanozyme-based electrochemical sensing system for ultrasensitive detection of African swine fever virus (ASFV) [61]. The Cu_2-X_S@Cu-MOF heterostructure exhibits significantly enhanced peroxidase-like activity compared to individual components, which is attributed to interfacial charge separation at the Cu_2-X_S/Cu-MOF junction and improved carrier mobility. Meanwhile, the porous Cu-MOF scaffold prevents nanoparticle aggregation and increases substrate accessibility for H_2_O_2_ and 3,3′,5,5′-tetramethylbenzidine (TMB), thereby amplifying catalytic efficiency. Importantly, the sensing platform integrates a dual-stage signal amplification strategy combining enzymatic TMB oxidation with electrochemical redox cycling, where each H_2_O_2_ molecule participates in multiple catalytic turnovers. This cascade amplification enables an exceptionally low detection limit of 6.45 × 10^−8^ TCID_50_/mL, demonstrating how nanozyme engineering and electrochemical signal recycling can synergistically push biosensing sensitivity toward the theoretical detection limit (Figure 4a) [61].

Beyond electrochemical amplification, magnetic enrichment combined with field-effect transistor (FET) readout represents another powerful strategy for real-time viral detection. As illustrated in Figure 4b, Liu et al. reported a Fe_3_O_4_@MIL-100-based magnetic MOF platform integrated with graphene field-effect transistors (GFETs) for SARS-CoV-2 detection [62]. In this system, the Fe_3_O_4_ core enables rapid magnetic separation and preconcentration of viral particles, while the MIL-100 shell is functionalized with virus-specific antibodies to ensure high selectivity. Following magnetic capture, the target viruses are directly transduced into electrical signals via GFETs, achieving an ultra-wide linear detection range from 1 ag/mL to 10 ng/mL and an exceptionally low detection limit of 8.60 ag/mL. This design highlights the importance of decoupling “target enrichment” and “signal transduction” into two modular steps, thereby significantly enhancing analytical sensitivity in complex biological matrices (Figure 4b) [62].

In addition to electrochemical and electronic readouts, optical biosensing strategies based on molecular imprinting and dual-signal amplification have also been developed to improve detection robustness. As shown in Figure 4c, Tang et al. constructed a molecularly imprinted virus sensor for enterovirus 71 (EV71) detection using Fe_3_O_4_@CDs magnetic carriers combined with aptamer-enhanced recognition elements [63]. The system integrates a two-stage “explosive” amplification mechanism: initial fluorescence quenching of carbon quantum dots upon target binding, followed by a secondary colorimetric signal amplification triggered by pH-induced decomposition of ZIF-8 and release of phenolphthalein. This dual-modal readout enables both fluorescence-based quantitative detection and naked-eye visual identification, achieving detection limits as low as 8.33 fM. Notably, the incorporation of molecular imprinting combined with aptamer recognition significantly enhances specificity, overcoming a key limitation of conventional imprinting systems in biologically complex environments (Figure 4c) [63].

Moving toward programmable and nucleic-acid-guided diagnostics, CRISPR-based biosensing platforms have emerged as a highly flexible and universally adaptable detection strategy. As shown in Figure 4d, Mao et al. developed a Pt@MOF-assisted CRISPR-Cas12a biosensing system for norovirus detection [64]. In this platform, Pt@MOF functions as both a signal transducer and catalytic enhancer, enabling dual-modal fluorescence and colorimetric readouts through base deprotonation-triggered cleavage of fluorogenic substrates. Importantly, integration of recombinase polymerase amplification (RPA) with noncanonical protospacer adjacent motif (PAM)-engineered CRISPR significantly improves sensitivity while expanding target recognition flexibility. This system achieves accurate detection in spiked food samples with 100% agreement with RT-qPCR, demonstrating its translational potential for real-world pathogen surveillance (Figure 4d) [64].

Taken together, these studies demonstrate that MOF-based biosensors can achieve remarkable analytical sensitivity through catalytic amplification, target enrichment, and signal transduction engineering. However, analytical performance should not be evaluated solely on the basis of detection limits. Many reported platforms have been validated primarily under controlled laboratory conditions, whereas comprehensive assessments of reproducibility, long-term stability, matrix tolerance, and large-scale clinical validation remain comparatively limited. In addition, ultra-low detection limits do not necessarily translate into improved clinical utility if assay complexity, turnaround time, or operational requirements restrict deployment in real-world settings. Future development should therefore emphasize balanced optimization of sensitivity, specificity, robustness, and practical applicability, together with standardized benchmarking protocols that enable meaningful comparisons across different biosensing platforms.

## 4. Host-Directed Antiviral Intervention

### Immune Modulation and Therapeutic Reprogramming

Unlike direct virucidal strategies that rely on external chemical or physical destruction of pathogens, host-directed antiviral interventions aim to reprogram the host immune system and reshape the disease microenvironment to achieve sustained antiviral efficacy. In this context, metal–organic frameworks (MOFs) have emerged as multifunctional immunomodulatory platforms capable of integrating controlled drug delivery, immune activation, and cellular reprogramming within a single engineered system. It should be noted that the biological outcomes discussed in this section primarily reflect host immune modulation and therapeutic responses rather than direct measurements of viral inactivation.

**Figure 5:**
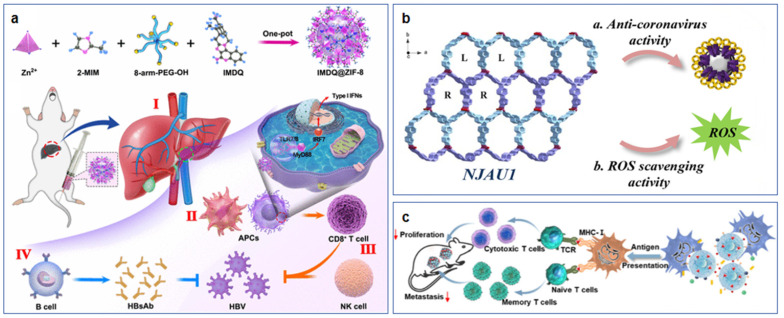
MOF-based host-directed antiviral intervention and immunomodulatory therapeutic platforms. (**a**) Liver-targeted delivery and immune activation cascade enabled by IMDQ@ZIF-8 nanoparticles for hepatitis B virus (HBV) immunotherapy, including antigen-presenting cell (APC) activation, downstream T cell and natural killer (NK) cell stimulation, and antibody-mediated viral clearance [65]. (**b**) Multifunctional zinc-based MOF (NJAU1) exhibiting broad-spectrum antiviral activity against coronaviruses through coordinated mechanisms, including viral inactivation, inhibition of viral entry and replication, and regulation of intracellular redox homeostasis via ROS scavenging and Zn^2+^-mediated modulation of ferroptosis and apoptosis pathways [66]. (**c**) Engineered macrophage-based immunotherapeutic strategy promoting enhanced phagocytosis of tumor cells, improved antigen presentation, and robust activation of tumor-specific CD8+ T cell responses, enabling strengthened adaptive anti-tumor immunity [67].

As shown in Figure 5a, Wang et al. developed a zeolitic imidazolate framework-8 (ZIF-8)-based nanocarrier for the delivery of an imidazoquinoline Toll-like receptor 7/8 (TLR7/8) agonist (IMDQ) for hepatitis B virus (HBV) immunotherapy [65]. A key limitation of free TLR agonists is their rapid systemic dispersion, which leads to off-target immune activation and significant toxicity. Encapsulation within ZIF-8 nanoparticles (IMDQ@ZIF-8 NPs) enables preferential liver accumulation and selective uptake by antigen-presenting cells (APCs), thereby spatially restricting immune activation to the desired immunological niche. This targeted delivery significantly enhances APC maturation and downstream adaptive immune responses, ultimately promoting robust viral clearance. Notably, treated HBV-infected mice exhibited increased anti-HBs antibody production and seroconversion, indicating successful restoration of functional antiviral immunity (Figure 5a) [65]. This work highlights how MOFs can act not only as passive carriers but also as immune “focusing systems” that spatially and temporally control immunostimulatory signaling.

Beyond pathogen-specific immunotherapy, MOF-based platforms have also been employed to modulate systemic antiviral microenvironments through redox and metabolic regulation. As illustrated in Figure 5b, Li et al. reported a zinc-based MOF (NJAU1) with a highly interconnected helical porous architecture, exhibiting broad-spectrum antiviral activity against multiple coronaviruses [66]. Mechanistically, NJAU1 operates through a multifaceted antiviral network involving direct viral particle disruption, inhibition of viral entry and replication, and regulation of intracellular redox homeostasis. Importantly, the material exhibits ROS-scavenging activity under pathological oxidative stress conditions, thereby restoring cellular redox balance while simultaneously suppressing virus-induced oxidative damage. In addition, Zn^2+^ release contributes to the regulation of ferroptosis and apoptosis pathways, further modulating host cell fate decisions during infection. This integrated functionality demonstrates that MOFs can simultaneously act as antiviral agents and cytoprotective regulators, rather than functioning solely as pathogen-targeting materials (Figure 5b) [66].

Moving toward cell-based immunotherapeutic engineering, MOFs have also been integrated into living immune cells to enhance antitumor antiviral-like immune responses. As shown in Figure 5c, Wang et al. developed engineered macrophages conjugated with oncolytic adenovirus-loaded ZIF-8 (ZIFOA-M) via bioorthogonal chemistry [22,67]. This hybrid platform enables localized viral replication within the tumor microenvironment, which downregulates “don’t eat me” signals such as CD47 and CD24 on tumor cells, thereby restoring macrophage phagocytic activity. In parallel, virus-induced immunogenic cell death leads to the release of damage-associated molecular patterns (DAMPs), which sustain M1-type macrophage polarization and amplify antigen presentation. Consequently, strong tumor-specific CD8^+^ T cell responses are elicited, resulting in durable adaptive immune activation (Figure 5c) [67]. This strategy highlights a highly advanced design paradigm in which MOFs function as interfaces between virotherapy and innate immune engineering, enabling multiscale immune system reprogramming.

Collectively, the studies presented in Figure 5 demonstrate a clear evolution of MOF-based host-directed antiviral strategies, progressing from passive drug delivery systems toward dynamic immunological regulators and ultimately toward engineered living-cell platforms. This transition underscores a central design principle: MOFs are increasingly serving as programmable immunomodulatory scaffolds that bridge nanomaterial engineering, virotherapy, and systems-level immune control for next-generation antiviral interventions. Importantly, many host-directed antiviral strategies are evaluated through immunological and cellular response indicators, which should be interpreted separately from direct measurements of viral infectivity or virucidal efficacy.

## 5. Virus-Inspired Therapeutic Platforms

### Viral Biomimicry and Delivery

Virus-inspired engineering represents a rapidly emerging paradigm in which metal–organic frameworks (MOFs) are no longer treated as passive carriers but are instead designed as structurally and functionally biomimetic systems that emulate viral architecture, infection pathways, and intracellular delivery mechanisms. By integrating hierarchical structural design with stimuli-responsive release behavior, MOF-based virus-mimetic platforms enable efficient transport across biological barriers and precise intracellular cargo delivery, thereby overcoming key limitations of conventional nanocarriers.

**Figure 6:**
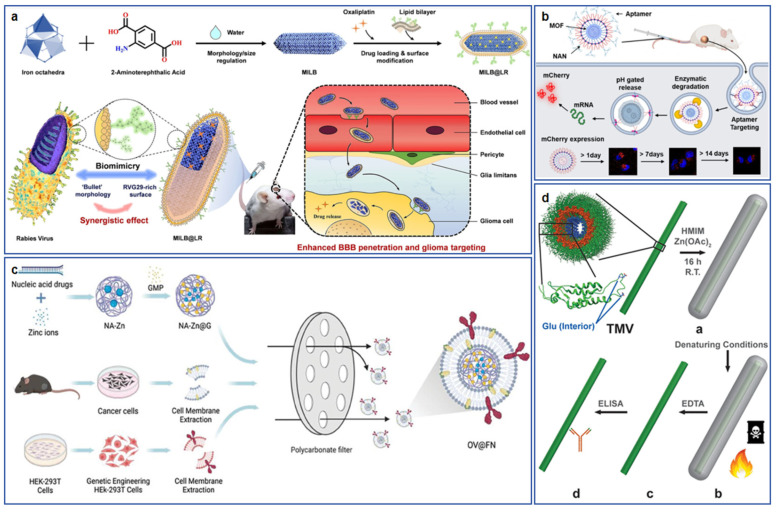
Virus-inspired and biomimetic MOF platforms for advanced drug delivery, gene therapy, and biointerface engineering. (**a**) Rabies virus (RABV)-inspired metal–organic framework nanocarrier (MILB@LR) fabricated with bullet-shaped architecture and surface-functional ligand decoration, enabling enhanced blood–brain barrier (BBB) penetration and targeted glioma therapy through multivalent viral-mimetic design principles [68]. (**b**) Virus-mimicking liposomal metal–organic framework nucleic acid nanocapsule for mRNA delivery, featuring a multilayered architecture with pH/enzyme-responsive gating that enhances intracellular delivery efficiency, improves mRNA stability, and enables receptor-specific expression in vitro and in vivo [69]. (**c**) Construction of oncolytic virus-like nanoparticles (OV@FN) integrating viral fusion membrane proteins with nanocarrier cores, enabling pH-responsive tumor targeting, cytoplasmic delivery of nucleic acids, and enhanced gene therapy efficacy through virus-mimetic membrane fusion mechanisms [70]. (**d**) Surface engineering and stability regulation of tobacco mosaic virus (TMV)-based MOF hybrid systems (TMV@ZIF), illustrating MOF encapsulation, stress-induced structural modulation, and chemically triggered recovery processes used to evaluate viral surface integrity and structural robustness under harsh conditions [71].

As shown in Figure 6a, Qiao et al. developed a rabies virus (RABV)-inspired MOF nanocarrier (MILB@LR) designed to overcome the blood–brain barrier (BBB), one of the most significant physiological obstacles in central nervous system (CNS) drug delivery [68]. By simultaneously mimicking the bullet-shaped morphology and surface functional characteristics of RABV, the engineered MILB@LR system achieves enhanced BBB penetration efficiency and selective accumulation in glioma tissues. Unlike conventional nanocarriers that rely on single-feature biomimicry, this integrated virus-mimetic strategy combines structural and interfacial engineering, resulting in improved cellular uptake and tumor-targeting specificity. When loaded with the chemotherapeutic agent oxaliplatin, MILB@LR significantly suppresses tumor growth in glioblastoma models, demonstrating the translational potential of virus-inspired MOF architectures for brain-targeted therapy (Figure 6a) [68]. This study highlights the importance of multi-dimensional viral mimicry in overcoming complex biological barriers such as the BBB.

Beyond structural biomimicry, virus-inspired MOF systems have also been extended to nucleic acid delivery platforms with enhanced stability and programmable release behavior. As illustrated in Figure 6b, Pal et al. reported a multilayered liposomal metal–organic framework nucleic acid nanocapsule for mRNA delivery [69]. In this system, a liposomal core is stabilized by an MOF shell and further encapsulated within a virus-mimicking hierarchical structure that responds to both pH and enzyme-specific triggers. This design reproduces a key biological feature of viral infection, in which endosomal acidification and enzymatic degradation trigger genome release. The resulting platform significantly improves mRNA stability during storage, maintains structural integrity for over 100 days at −20 °C, and enables sustained protein expression in vitro and in vivo. Moreover, receptor-mediated targeting enables more homogeneous expression within tumor microenvironments, demonstrating that MOF-based virus mimics can extend beyond delivery efficiency to regulate spatial gene expression profiles (Figure 6b) [69].

In addition to nucleic acid delivery, virus-mimetic strategies have also been employed to enhance intracellular gene transport and tumor-specific cytoplasmic delivery. As shown in Figure 6c, Li et al. constructed oncolytic virus-like nanoparticles (OV@FN) by integrating a nano-core (NA-Zn@G) with a hybrid membrane vesicle expressing viral fusion glycoproteins [70]. This system leverages virus-like membrane fusion mechanisms to facilitate efficient cytoplasmic entry under mildly acidic tumor microenvironment conditions. Once internalized, the nano-core responds to high intracellular glutathione levels to release nucleic acid payloads in a controlled manner, ensuring spatially and temporally precise gene expression. Importantly, the engineered viral membrane component promotes tumor cell syncytium formation, enhancing intercellular diffusion of therapeutic cargo and amplifying gene therapy efficacy. In vivo studies further demonstrate strong tumor suppression and immunomodulatory effects in melanoma models, highlighting the multifunctional therapeutic potential of virus-like MOF platforms (Figure 6c) [70]. Moving toward vaccine stabilization and structural preservation, MOF-based systems have also been applied to protect viral and proteinaceous structures under harsh environmental conditions. As illustrated in Figure 6d, Luzuriaga et al. investigated zeolitic imidazolate framework-8 (ZIF-8) for stabilizing viral vectors and protein-based vaccines [71]. The MOF encapsulation provides a rigid porous shell that preserves native conformations of viral particles and prevents denaturation under thermal and chemical stress. Immunological and spectroscopic analyses confirmed that the encapsulated viral nanoparticles retain long-term biological activity and immunogenicity. Furthermore, in vivo studies demonstrated excellent biocompatibility, with no observable tissue damage following repeated administration. This work establishes MOFs as effective protective matrices for biologics, enabling controlled release while maintaining structural integrity and functional stability in physiological environments (Figure 6d) [71].

Collectively, the studies presented in Figure 6 demonstrate a clear evolution in MOF-based virus-inspired platforms, progressing from simple structural mimicry toward multifunctional systems capable of integrating barrier penetration, stimuli-responsive cargo release, intracellular trafficking, and biomacromolecule stabilization. This convergence of biomimetic engineering and materials chemistry positions MOFs as a unique class of virus-like therapeutic systems that bridge nanomedicine, synthetic virology, and precision drug delivery.

## 6. Emerging Opportunities

### 6.1. MOF-Assisted Antiviral Drug Discovery

Beyond diagnostics and therapeutic delivery, metal–organic frameworks (MOFs) are increasingly emerging as enabling platforms for antiviral drug discovery, particularly in addressing the long-standing challenge of screening bioactive compounds from chemically complex natural product libraries. A representative advance is the MOF-enabled enzyme immobilization strategy reported by Zhao et al. [72], where influenza neuraminidase (NA)—a clinically validated antiviral target—was immobilized onto MOF-808 through a cross-linker-free in situ precipitation approach. This design exploits ammonium sulfate–induced precipitation to drive stable enzyme anchoring, while synergistic electrostatic and hydrophilic interactions ensure high enzyme loading capacity (19.1 mg·g^−1^) and retention of native enzymatic conformation [72].

Importantly, the moderately aggregated MOF-808 architecture forms a “protective cage” microenvironment that mitigates protein denaturation and preserves the functional integrity of NA, while simultaneously enhancing local substrate enrichment at catalytic sites [73,74,75]. This confined enzymatic microenvironment is conceptually aligned with the broader MOF “bio-interface engineering” paradigm, where porous coordination networks act not only as passive supports but as active regulators of biomolecular stability and reaction kinetics [76,77,78,79].

Leveraging this stabilized enzymatic platform, MOF-808@NA was further employed as an affinity-based screening medium for ligand fishing from the traditional Chinese medicine (TCM) formulation Mahuang–Xixin–Fuzi decoction. This approach enabled the identification of 12 candidate neuraminidase inhibitors via HPLC-QTOF-MS/MS analysis [80,81,82,83], demonstrating how MOF-confined enzymes can bridge chemical biology and natural product pharmacology in a high-throughput yet functionally relevant manner [84,85,86].

Functionally, the enriched fraction obtained from MOF-808@NA exhibited a 26-fold increase in NA inhibitory activity relative to the crude extract, along with a 2.4-fold enhancement in antiviral activity in H1N1-infected cellular assays [87,88,89]. These results highlight that MOF-based enzyme immobilization does not merely serve as a stabilization strategy, but actively amplifies the sensitivity and selectivity of bioactive molecule discovery by preserving native enzymatic recognition landscapes under complex chemical environments [90].

Collectively, this study exemplifies a shift from conventional “hit-or-miss” antiviral screening toward structure-guided, enzyme-confined discovery systems enabled by MOF microenvironments [91]. More broadly, it suggests that future MOF-assisted antiviral discovery platforms may evolve into integrated bio-catalytic screening reactors, capable of coupling molecular recognition, stabilization, and high-throughput screening within a single material system—thereby significantly accelerating the identification of next-generation antiviral lead compounds [92].

### 6.2. AI-Guided Anti-Pathogen MOFs

Recent progress in MOF-based biomedical applications has increasingly converged with artificial intelligence (AI)-driven materials design, enabling a transition from empirically optimized structures toward predictive, data-informed discovery frameworks. In the context of anti-pathogen systems, this paradigm shift is particularly significant, as MOFs must simultaneously satisfy multiple competing requirements, including biocompatibility, selective binding, stability in physiological environments, and tunable host–guest interactions.

A representative perspective is provided by Quan et al. [93], who systematically reviewed the emerging concept of oral MOFs for gastrointestinal (GI) detoxification, highlighting how computational and AI-assisted strategies can guide the rational design of functional coordination frameworks for in vivo applications. Although this work is not focused exclusively on antiviral systems, its conceptual implications extend directly to anti-pathogen MOF development, particularly in terms of structure–function optimization under complex biological conditions.

Specifically, AI-assisted modeling is increasingly being integrated to predict MOF adsorption behavior, stability across pH gradients in the GI tract, and interaction selectivity toward toxic molecules or microbial metabolites [93]. This is critical for translating MOFs from in vitro proof-of-concept systems to clinically relevant oral or systemic anti-pathogen platforms, where environmental heterogeneity often dictates performance failure. By incorporating physiological constraints—such as enzymatic degradation, microbiota-mediated transformation, and dynamic pH fluctuations—AI frameworks can help prioritize MOF compositions with robust in vivo resilience and targeted functionality.

Importantly, the review further emphasizes that MOF–microbiota interactions represent a bidirectional regulatory axis, where MOFs can modulate microbial communities while being simultaneously transformed by them [94]. From an anti-pathogen perspective, this introduces a higher-order design variable: the therapeutic efficacy of MOFs is no longer solely determined by intrinsic material properties, but also by emergent ecosystem-level interactions within the host environment. AI-guided approaches are therefore uniquely positioned to integrate multi-scale datasets—including materials descriptors, biological response profiles, and pharmacokinetic parameters—to optimize such complex systems [95].

Taken together, AI-guided MOF design represents a critical enabling technology for next-generation anti-pathogen materials, offering a route toward rational, rather than trial-and-error, development of functional coordination frameworks [96]. While still in its early stages, this approach is expected to significantly accelerate the discovery of clinically translatable MOF systems, particularly for oral and systemic applications where biological complexity remains a major barrier.

### 6.3. Clinical Translation Challenges

Despite the rapid expansion of metal–organic frameworks (MOFs) in antiviral, antibacterial, and broader anti-pathogen applications, their clinical translation remains constrained by a series of interconnected scientific, engineering, and regulatory challenges. As highlighted across recent reviews on functional inorganic antiviral materials, MOF-based biomedical systems must ultimately operate in complex and fluctuating physiological environments where stability, safety, and sustained efficacy become decisive factors beyond laboratory performance metrics [97].

From a materials chemistry perspective, one of the primary translational barriers is the long-term structural and chemical stability of MOFs under biologically relevant conditions. Many frameworks exhibit sensitivity to aqueous media, ionic strength, or enzymatic environments, which can lead to premature degradation or uncontrolled release of active components. Although such degradation can be advantageous in catalytic antiviral processes, as demonstrated in reactive oxygen species (ROS)-mediated inactivation pathways, it simultaneously raises concerns regarding dose control and off-target toxicity in vivo [97,98]. This duality reflects a broader challenge in designing MOFs that balance reactivity with structural persistence across different application contexts.

Another critical limitation lies in biosafety and immunological compatibility. While MOFs are often described as biocompatible platforms, their long-term interactions with immune systems, protein coronas, and metabolic pathways remain insufficiently characterized. Reviews on biomedical MOFs emphasize that in vivo applications—including drug delivery, imaging, and vaccination—require strict control over particle size, surface chemistry, and degradation products to minimize unintended immune activation or chronic accumulation [98,99]. These considerations become particularly important for nucleic acid delivery systems, where MOFs serve as non-viral vectors but must avoid inducing cytotoxicity or immunogenic responses comparable to viral carriers [99].

A further translational bottleneck arises from the gap between high-performance laboratory demonstrations and real-world deployment formats. As noted in recent analyses of MOF-based protective materials, including catalytic coatings and filtration systems, powder-based MOFs are often unsuitable for integration into scalable devices such as masks, textiles, or implantable platforms without additional engineering into robust composites or thin films. Similarly, while zirconium-based MOFs demonstrate rapid catalytic detoxification of chemical and biological threats, their practical implementation requires careful immobilization strategies to ensure mechanical stability and long-term functionality under operational conditions.

Finally, regulatory and manufacturing challenges represent a nontrivial barrier to clinical adoption. The heterogeneity of MOF compositions, coupled with the lack of standardized toxicity evaluation frameworks, complicates reproducibility and quality control across large-scale production. In parallel, the multifunctionality that makes MOFs attractive for anti-pathogen applications—such as combined catalytic, adsorptive, and delivery capabilities—also increases the complexity of regulatory approval pathways, particularly when transitioning from in vitro validation to in vivo or clinical use.

Collectively, these challenges highlight that successful clinical translation of MOF-based anti-pathogen systems will require a coordinated advancement in materials stability engineering, biointerface understanding, scalable fabrication technologies, and regulatory standardization. Addressing these issues will be essential to bridge the current gap between proof-of-concept studies and clinically deployable MOF-enabled therapeutic and protective platforms [97,98,99].

Importantly, the overall maturity of the field should be interpreted with caution. Although MOF-based anti-pathogen systems have demonstrated impressive performance in pathogen inactivation, biosensing, and therapeutic delivery, the majority of reported studies remain at the proof-of-concept stage. Most investigations have been conducted under simplified in vitro conditions or limited animal models, while systematic studies addressing pharmacokinetics, long-term biosafety, manufacturing reproducibility, and clinical efficacy are still scarce. Consequently, many current advances should be regarded as promising concepts rather than clinically mature technologies. Bridging this gap will require standardized evaluation protocols, interdisciplinary collaboration, and increased emphasis on translationally relevant studies.

Beyond individual applications, the studies summarized throughout this Review reveal several common structure–function relationships that may guide the development of next-generation anti-pathogen MOFs. Across different application scenarios, precise engineering of catalytic centers and coordination environments determines biological selectivity and reaction specificity, whereas hierarchical pore architectures and host–guest interactions govern molecular accessibility, cargo loading, and signal amplification. Equally important, surface and interface engineering—including functionalization with biomolecules, polymers, and biomimetic components—plays a critical role in biocompatibility, target recognition, and intracellular transport. These observations collectively indicate that future MOF design should increasingly emphasize the integration of catalytic activity, sensing capability, immune modulation, and therapeutic delivery within unified architectures. Such structure–function relationships provide a rational framework for developing more effective and clinically translatable anti-pathogen systems. As anti-pathogen MOF platforms become increasingly multifunctional and application-oriented, future development may also benefit from system-level optimization methodologies that integrate materials design, performance evaluation, translational considerations, and data-driven decision making. Similar trends have emerged across other complex technological domains, where artificial intelligence-assisted optimization, systems-level governance, and interdisciplinary innovation frameworks are increasingly used to accelerate technology deployment and improve decision efficiency [100,101,102].

## 7. Conclusions

Recent advances have expanded the role of metal–organic frameworks (MOFs) from conventional antimicrobial materials to multifunctional anti-pathogen platforms. As summarized in this Review, MOF-based systems have been explored for direct pathogen inactivation, biosensing, host-directed intervention, and virus-inspired therapeutic delivery. In the area of pathogen inactivation, MOF-based antiviral strategies have evolved beyond conventional reactive oxygen species (ROS)-mediated oxidation toward more selective mechanisms involving enzyme-mimicking catalysis, protein denaturation, and membrane-targeted disruption. In parallel, the integration of catalytic amplification, magnetic enrichment, electrochemical transduction, and CRISPR-based recognition has facilitated the development of highly sensitive diagnostic platforms. Furthermore, host-directed immunomodulation and virus-inspired delivery systems have expanded the functional scope of MOFs from pathogen elimination to therapeutic regulation and intracellular cargo transport.

Despite these advances, several challenges remain before practical and clinical translation can be achieved. Long-term structural stability under physiological conditions, degradation behavior, biodistribution, biosafety evaluation, and scalable manufacturing require further investigation. In addition, standardized assessment protocols and the development of clinically relevant formulations and devices will be essential for improving reproducibility and translational feasibility.

Future research may further explore precision catalytic design, integrated diagnostic–therapeutic platforms, and artificial intelligence-assisted materials discovery, although the clinical feasibility of these approaches remains to be established. Continued efforts to understand material–biological interactions and address translational challenges are expected to facilitate the development of safer, more effective, and clinically relevant MOF-based anti-pathogen technologies.

## Data Availability

No data sharing statement is applicable for this article, as it does not involve the generation of new data.
